# Metabolic engineering of *Pseudomonas* sp. strain VLB120 as platform biocatalyst for the production of isobutyric acid and other secondary metabolites

**DOI:** 10.1186/1475-2859-13-2

**Published:** 2014-01-07

**Authors:** Karsten Lang, Jessica Zierow, Katja Buehler, Andreas Schmid

**Affiliations:** 1 Department of Biochemical and Chemical Engineering, Laboratory of Chemical Biotechnology, TU Dortmund University, Emil-Figge-Strasse 66, 44227 Dortmund, Germany

**Keywords:** Isobutyric acid, Isobutanol, Pseudomonas, Fermentative, Valine synthesis route

## Abstract

**Background:**

Over the recent years the production of Ehrlich pathway derived chemicals was shown in a variety of hosts such as *Escherichia coli*, *Corynebacterium glutamicum*, and yeast. Exemplarily the production of isobutyric acid was demonstrated in *Escherichia coli* with remarkable titers and yields. However, these examples suffer from byproduct formation due to the fermentative growth mode of the respective organism. We aim at establishing a new aerobic, chassis for the synthesis of isobutyric acid and other interesting metabolites using *Pseudomonas* sp. strain VLB120, an obligate aerobe organism, as host strain.

**Results:**

The overexpression of *kivd*, coding for a 2-ketoacid decarboxylase from *Lactococcus lactis* in *Ps*. sp. strain VLB120 enabled for the production of isobutyric acid and isobutanol via the valine synthesis route (Ehrlich pathway). This indicates the existence of chromosomally encoded alcohol and aldehyde dehydrogenases catalyzing the reduction and oxidation of isobutyraldehyde. In addition we showed that the strain possesses a complete pathway for isobutyric acid metabolization, channeling the compound via isobutyryl-CoA into valine degradation. Three key issues were addressed to allow and optimize isobutyric acid synthesis: i) minimizing isobutyric acid degradation by host intrinsic enzymes, ii) construction of suitable expression systems and iii) streamlining of central carbon metabolism finally leading to production of up to 26.8 ± 1.5 mM isobutyric acid with a carbon yield of 0.12 ± 0.01 g g_glc_^-1^.

**Conclusion:**

The combination of an increased flux towards isobutyric acid using a tailor-made expression system and the prevention of precursor and product degradation allowed efficient production of isobutyric acid in *Ps*. sp. strain VLB120. This will be the basis for the development of a continuous reaction process for this bulk chemicals.

## Background

The finite nature of fossil resources necessitates the development of new technologies for the production of chemicals based on natural renewable feedstocks. So far, approximately 90% of all chemicals are produced from fossil-based supplies, but the world market for bio-based chemicals is expected to increase from $US 3.6 billion in 2011 to $US 12.2 billion in 2021 [1-3]. The rapid progress in the fields of metabolic engineering and systems biology accelerates this development and allows the synthesis of non-natural and non-inherent products [4]. Atsumi et al. [5] showed the production of higher alcohols via the amino acid catabolism by integrating the Ehrlich pathway into *Escherichia coli*. 2-keto acid intermediates were decarboxylated to the corresponding aldehydes using the 2-keto acid decarboxylase Kivd from *Lactococcus lactis* and were further converted by (host intrinsic) alcohol dehydrogenases to higher alcohols. Zhang et al. [6] demonstrated the successful oxidation of isobutyraldehyde to isobutyric acid in *E. coli*, by overexpressing an aldehyde dehydrogenase. Isobutyric acid is mainly used as a precursor for methacrylic acid production and has a market size of about 2.7 ∙ 10^6^ t a^-1^. In addition, isobutyric acid can be utilized for the production of sucrose acetoisobutyrate, texanol or di-isobutyrate [7]. Isobutyric acid can be chemically produced by reacting propene, carbon monoxide, and water in the presence of strong acids [8]. This chemical route suffers from its dependency on fossil products of oil refining and natural gas processes and the involvement of compounds harmful to the environment such as sulfuric acid, hydrogen fluoride, and boron fluoride [6].

A major bottleneck of current bioprocesses is the susceptibility of microorganisms to toxic compounds [9,10]. This results in low product titers and limited process stabilities having a negative impact on overall productivity. The cost competitiveness of biocatalytic processes is determined by these parameters [11] and it is indispensable to overcome these limitations. *Pseudomonas* species are known to exhibit mechanism enabling the adaptation to toxic environmental conditions [12]. Importantly, these organisms are able to form stable surface associated microbial communities (biofilms), which have been described as potent alternative to planktonic cells regarding their application as biocatalyst, especially when solvents or otherwise toxic compounds are involved in the processes [13]. Biofilms provide increased process stability and therefore potentially allow, in contrast to commonly used planktonic cells, a continuous process mode [14,15].

Pseudomonads have a huge potential for bioremediation, and possess a rich pathway repertory for the degradation of a variety of non-natural and non-inherent toxic compounds such as aromatic organics [16-19]. Carbon is utilized in *Pseudomonas* wild types almost without the production of byproducts such as acetate [20], lactate, glycerol or ethanol, which improves carbon yields and simplifies downstream processing in comparison to organisms like *Escherichia coli*, *Bacillus subtilis*, and *Saccharomyces cerevisiae*[21]. Under anaerobic and micro aerobic conditions, *E. coli* needs to produce mixed-acids directly from the key-precursor pyruvate to maintain an optimal redox and carbon environment for isobutyric acid synthesis, finally limiting the final carbon yield and affecting the downstream processing. In aerobic environment *E. coli* is known for its overflow metabolism under glucose excess conditions resulting in acetate formation [22].

So far, mainly aromatic compounds are produced using recombinant *Pseudomonas* strains. The fermentative *de-novo* synthesis of e.g. phenol, *p*-coumarate, *p*-hydroxystyrene, *t*-cinnamate, and several polyhydroxyalkanoates [9,23-27] from glucose have been reported as well as biotransformations using toluene or styrene for the production of 3-methylcatechol and *(S)*-styrene oxide [28,29]. Moreover, Nielsen et al. [30] reported the fermentative production of 1-butanol using an engineered *Pseudomonas putida* S12. Here the term ‘fermentation’ describes synthesis of the target product by whole cells directly from the added carbon and energy source, whereas ‘biotransformation’ refers to processes, where in addition to the growth substrate a biotransfomation substrate is added [31].

This study aims to establish the solvent tolerant, genome-sequenced *Pseudomonas* sp. strain VLB120 [29,32] as a platform biocatalyst for the fermentative production of isobutyric acid. Therefore the Ehrlich pathway is incorporated into a Pseudomonad with the aim to produce isobutyric acid via the valine synthesis pathway. In this work, strain specific limitations of *Pseudomonas* like low expression levels of recombinant genes and product and precursor degradation were solved by means of metabolic engineering.

## Results

### Overexpression of a 2-keto acid decarboxylase encoding gene in *Pseudomonas* sp. strain VLB120 allows the synthesis of isobutyric acid and isobutanol

To evaluate the application of *Ps.* sp. strain VLB120 for the synthesis of isobutyric acid, the 2-keto acid decarboxylase Kivd from *Lactococcus lactis* was produced using a pCOM10 expression system. The resulting *Ps.* sp. strain VLB120 pCOM10-*kivd* produced isobutyric acid, isobutanol, and 3-methyl-1-butanol directly from glucose (Table 1). The presence of isobutanol and isobutyric acid indicate the existence of host intrinsic alcohol and aldehyde dehydrogenases in *Ps.* sp. strain VLB120 capable to oxidize or reduce isobutyraldehyde. In abiotic experiments no isobutyric acid formation could be detected. Product titers were significantly increased by the addition of yeast extract, which can serve as carbon precursor for the desired products. The production of 3-methyl-1-butanol can be explained by a withdrawal of 2-keto-isocaproate, the precursor of leucine biosynthesis, which is first decarboxylated and then reduced by host intrinsic enzymes to the corresponding alcohol [33].

**Table 1:** Influence of yeast extract on product titers of isobutyric acid, isobutanol and 3-methyl-1-butanol

	**Isobutyric acid [μM]**	**Isobutanol [μM]**	**3-methyl-1-butanol [μM]**
M9*	5.4 ± 1.3	43.1 ± 0.4	50.0 ± 1.4
M9* + 5 g L^-1^ yeast extract	56.4 ± 1.5	72.1 ± 2.9	236.5 ± 0.4

Cultivations (72 h) were done in M9* medium with 20 g L^-1^ glucose in sealed shake flasks at 30°C with and without 5 g L^-1^ yeast extract using *Pseudomonas* sp. strain VLB120 pCOM10-*kivd. kivd* overexpression was induced with 0.05% (v/v) DCPK at early exponential phase. In cultures of *Pseudomonas* sp. strain pCOM10 without overexpression of *kivd* none of the listed products were detectable. Errors indicate standard deviations (n = 2).

### Product and precursor degradation in *Pseudomonas* sp. strain VLB120

*Pseudomonas* species are known for their ability to utilize a variety of organic molecules as growth substrates [16]. This ability may contradict process efficiency by product or precursor degradation. To elucidate whether the desired product and/or precursors are degraded, growth experiments, using the respective compounds as sole carbon source were conducted (Table 2). *Ps.* sp. strain VLB120 is able to grow on all tested compounds with different carbon yields and growth rates. After pre-growth in the presence of the respective substrates to allow cell metabolism to adapt, isobutyric acid, isobutyraldehyde, and 2-ketoisovalerate (2-KIV) were converted allowing exponential growth rates between of 0.15 and 0.24 h^-1^, with the exception of isobutanol where only slow growth was observed. Biomass yields from glucose for isobutyric acid, and isobutyraldehyde are in the same range, while conversion of 2-KIV led to lower final biomass concentrations.

**Table 2:** **Growth rate (μ) and biomass yield (Y) of *Pseudomonas *** **sp. strain VLB120 on different carbon sources**

**Carbon source**	**μ (h**^**-1**^**)**	**Y (g**_**cdw**_**/g**_**carbon source**_**)**
Glucose	0.41 ± 0.01	0.64 ± 0.01
2-KIV	0.24 ± 0.03	0.39 ± 0.05
Isobutyraldehyde	0.24 ± 0.01	0.52 ± 0.01
Isobutyric acid	0.15 ± 0.02	0.59 ± 0.04
Isobutanol	0.025 ± 0.001	n.d.

Cells were cultivated in sealed shake flasks at 30°C on 10 mM of different carbon sources in M9* medium pH 7.0. Errors indicate standard deviations (n = 2 - 5).

n.d.: not determined due to very slow growth (long time experiments result in errors caused by oxygen limitation and/or isobutanol evaporation).

**Figure 1:**
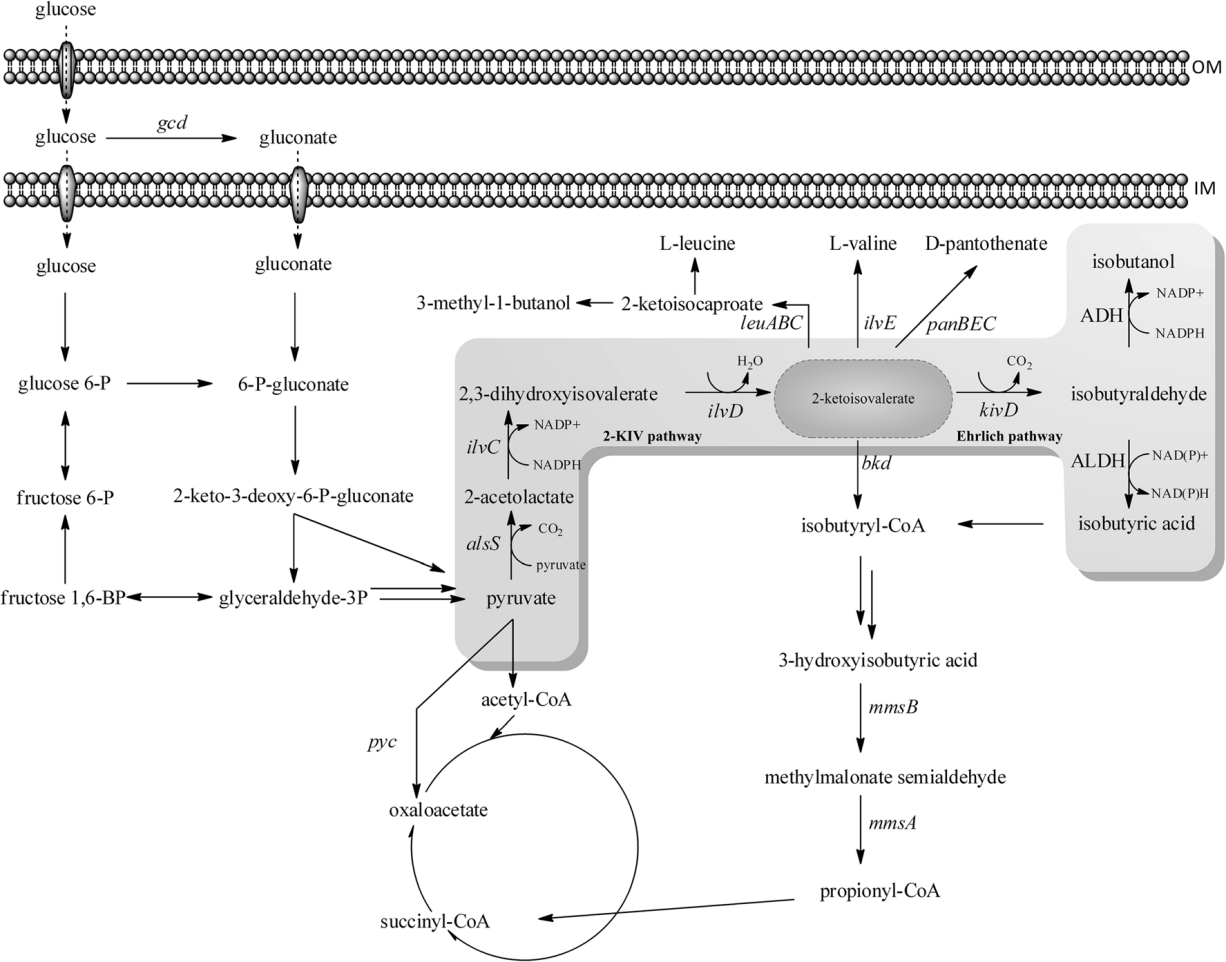
**Pathways for isobutyric acid synthesis and its degradation in *Ps*. sp. strain VLB120.** OM: outer membrane, IM: inner membrane, ADH: alcohol dehydrogenase, ALDH: aldehyde dehydrogenase, *alsS*: acetolactate synthase, *bkd*: branched-chain alpha-keto acid dehydrogenase gene cluster*, gcd*: glucose dehydrogenase, *ilvC*: ketol-acid reductoisomerase, *ilvD*: dihydroxy acid dehydratase, *ilvE*: branched-chain-amino-acid transaminase, *kivd*: 2-keto acid decarboxylase, *leuA*: 2-isopropylmalate synthase, *leuB*: 3-isopropylmalate dehydrogenase*, leuC*: isopropylmalate isomerase, *mmsA*: methylmalonate-semialdehyde dehydrogenase, *mmsB*: 3–hydroxyisobutyrate dehydrogenase, *pyc*: pyruvate carboxylase, *panB*: ketopantoate hydroxymethyl transferase, *panE*: ketopantoic acid reductase, *panC* pantothenate synthetase [19,40,53].

To optimize isobutyric acid production precursor and product degradation needed to be prevented or at least minimized. Based on the growth experiments and genomic data a map was constructed showing the overall pathways for isobutyric acid synthesis and its degradation in *Ps.* sp. strain VLB120 using glucose as carbon source (Figure 1).

### Deletion of branched chain 2-keto acid dehydrogenase (*bkd*) strongly increases product titers in 2-KIV biotransformations

**Figure 2:**
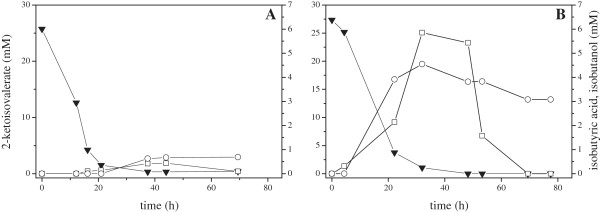
**Growing cell 2-ketoisovalerate biotransformations.** Comparison of *Ps.* sp. strain VLB120 pCOM10-*kivd***(A)** and *Ps.* sp. strain VLB120Δ*bkd* pCOM10-*kivd***(B)**. Cultivations were performed in M9* pH 7.0, 5 g L^-1^ glucose in sealed shake flasks at 30°C. Cells were induced at early exponential phase with 0.05% (v/v) DCPK and 25 mM 2-KIV was added. ▼: 2-ketoisovalerate, □: isobutyric acid, ○: isobutanol. Two independent fermentations were performed, showing comparable results.

The degradation of valine was extensively investigated in *P. putida* and it was demonstrated that a branched chain α-keto acid dehydrogenase complex consisting of three proteins is responsible for the oxidation of 2-KIV to isobutyryl-CoA, which is further degraded to propionyl-CoA [34,35] (Figure 2). In the genome of *Ps.* sp. strain VLB120, a homolog of this gene cluster (PVLB_17450, PVLB_17455, PVLB_17460 and PVLB_17465) was identified. To prevent the degradation of 2-KIV the gene coding for the E1 subunit of the branched chain 2-keto acid dehydrogenase (PVLB_17450) was deleted. The resulting Δ*bkd* strain could not grow on 2-KIV as sole carbon source anymore (data not shown). Degradation of 2-KIV was investigated in Ps. sp. strain VLB120 Δ*bkd* and the wild type strain, both harboring pCOM10-*kivd* (Figure 2). The mutant showed a lower degradation rate of 2-KIV, whereas the production titer of isobutanol and isobutyric acid increased significantly. Isobutyric acid accumulated over the first 30 h and was metabolized completely over the next 40 h, while isobutanol was again degraded at a slow rate (Figure 2, see also Table 2).

### Random mutagenesis to prevent isobutyric acid degradation

**Figure 3:**
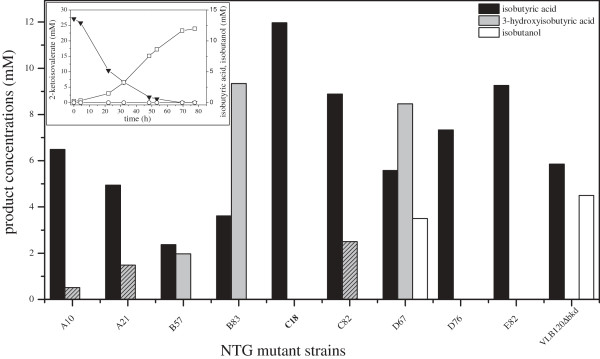
**Maximum achievable product titers of isobutyric acid, 3-hydroxyisobutyric acid, and isobutanol during 2-ketoisovalerate biotransformations.** Growing *Ps.* sp. strain VLB120 NTG mutants transformed with pCOM10-*kivd* were utilized. Exemplarily concentration profiles of 2-KIV, isobutanol and isobutyric acid in the NTG mutant *Ps.* sp. strain VLB120 C18 are highlighted in the upper left box. Cultivations were performed in M9* pH 7.0, 5 g L^-1^ glucose in sealed shake flasks at 30°C. Cells were induced at early exponential phase with 0.05% (v/v) DCPK. At the same time point 25 mM 2-KIV was added. Hatched bars denote concurrent product degradation. ▼: 2-ketoisovalerate, □: isobutyric acid, ○: isobutanol. (n = 1, due to expensive substrate).

Isobutyric acid is a carbon and energy source for *Pseudomonas* species and knowledge about the enzymes responsible for its degradation is limited. Godhue et al. [36] showed the whole-cell biotransformation of isobutyrate to L-(+)-3-hydroxyisobutyrate in *P. putida* ATCC21244. This indicates the existence of an enzyme activity catalyzing the reaction from isobutyric acid to isobutyryl-CoA, which is a metabolite in the natural valine degradation pathway. Random mutagenesis of *Ps*. sp. strain VLB120Δ*bkd* using *N*-methyl-*N*’-nitro-*N*-nitrosoguanidine (NTG) was performed to mutate the respective genes. NTG is one of the most widespread chemical mutagens, and well known for its ability to create auxotrophic mutants [37]. In *Pseudomonas* species, it was used amongst others to optimize cinnamic acid and phenol production [9,38]. The optimal compromise between cell survival rate and auxothrophy frequency was found at a NTG concentration of 80 μg mL^-1^, which is comparable to other reports [9,39]. We modified a method, which was used by Martin et al. [40] to create mutants of the valine degradation pathway. Mutants showing no alteration in growth on glucose and succinate but a drastically decreased growth rate and/or *lag* phase on isobutyric acid, were selected for further investigation (Additional file 1: Figure S1). 2-KIV biotransformations were performed with NTG-mutants harboring pCOM10-*kivd* (Figure 3). All mutants showed a change in their 2-KIV conversion rate. Some mutants (A10, A21, B57, B83, C82, D67) accumulated 3-hydroxyisobutyric acid and isobutyric acid, others only isobutyric acid (C18, D76, E82). Surprisingly, all mutants except D67 lost their ability to accumulate isobutanol even temporarily under these conditions. This product was only observed in resting cell assays, when 10 mM isobutyraldehyde were applied as substrate (Additional file 1: Figure S2). Mutant C18 was chosen as the candidate for further strain development, as it produced isobutyric acid with the highest yield compared to *Ps.* sp. strain VLB120Δ*bkd* and no product degradation could be detected.

In general, 2-KIV biotransformations showed a maximum yield of approximately 50% isobutyric acid from 2-KIV. This compound serves as a precursor for leucine, valine and panthothenic acid synthesis which negatively influences the final yield (Figure 1).

### Combined T7 overexpression of valine synthesis and Ehrlich pathway genes showed increased enzyme activity for each single protein

To increase the flux to 2-KIV, the respective genes of the valine synthesis pathway were overexpressed in *Ps.* sp. strain VLB120 T7. This strain was developed to guarantee stable and high expression levels in the desired host under fermentative conditions. T7 promoter dependent expression turned out to be beneficial, as compared to P_alk_-dependent expression (Additional file 1: Figure S2). *Ps*. sp. strain VLB120 T7 was transformed with plasmids pPAPC-Km and pPDPK. For details on plasmid construction please see materials and methods. Activities for AlsS, IlvC, IlvD and Kivd were determined in crude cell extracts, and compared to activities in extracts of the wild type and *Ps*. sp. VLB120 pCOM10-*kivd* (Table 3).

**Table 3:** **Specific activities of AlsS, IlvC, IlvD, Kivd in cell extracts of *Pseudomonas *** **sp. strain VLB120, *Pseudomonas *** **sp. strain T7 pPAPC-Km, pPDPK and *Ps. *** **sp. strain VLB120 pCom10-kivd**

	**Specific activity (μmol min**^**-1 **^**mg**_**total protein**_^**-1**^**)**			
	AlsS	IlvC	IlvD	Kivd
VLB120 wild type	0.033 ± 0.009	0.0257 ± 0.0017^*^	0.0075 ± 0.0004	ND
VLB120 T7 pPAPC-Km, pPDPK	3.494 ± 0.299	0.1476 ± 0.0425^*^	0.1955 ± 0.0023	2.96 ± 0.24
VLB120 pCOM10-*kivd*	n.d.	n.d.	n.d.	ND

ND: not detectable.

^*^: coupled assay together with AlsS.

Cells were cultured in M9* pH 7.4, 20 g L^-1^ glucose and. 5 g L^-1^ yeast extract shake flasks at 30° Cells were induced for 4 h either with 0.05% DCPK (pCOM10) or with 1 mM IPTG (pPDPK, pPAPC-Km) in the early exponential phase. Errors indicate standard deviations (n = 2 - 3).

All proteins showed higher specific activities in the desired production host as compared to the wild type strain. Under the fermentation conditions used, Kivd activity in *Ps.* sp. strain pCOM10-*kivd* was not even detectable, being in line with the eGFP expression study (Additional file 1: Figure S2), and demonstrated the advantage of T7 expression in *Ps*. sp. strain VLB120.

### Overexpression of the 2-KIV and Ehrlich pathway in the NTG mutant C18 T7 results in stable isobutyric acid production

**Figure 4:**
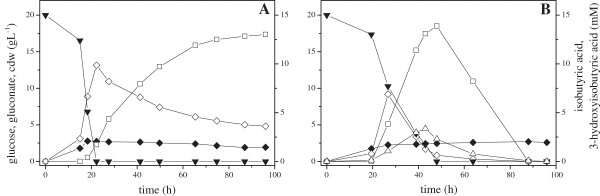
**Isobutyric acid production during a representative fermentation.***Ps.* sp. strain VLB120 T7. pPAPC-Km, pPDPK **(A)** and C18 T7 pPAPC-Km, pPDPK **(B)** were cultivated in M9* pH 7.4 medium initially containing 20 g L^-1^ glucose and 5 g L^-1^ yeast extract. Cells were induced with 1 mM IPTG at timepoint 0 h at an OD_450_ of 10. ▼: glucose, ◊: gluconate, ♦: biomass concentration, □: isobutyric acid, ○: isobutanol, Δ: 3-hydroxyisobutyric acid. Three independent fermentations were performed, all showing comparable results.

2-KIV and Ehrlich pathway overexpression in *Ps.* sp. strain VLB120 T7 (Figure 4A) and in the genetic engineered variant C18 T7 (Figure 4B) resulted in drastically increased final isobutyric acid titers in aerobic fermentations. Shake flask experiments were carried out with carbon concentrations of 20 g L^-1^ glucose to avoid a pH drop. Cells were induced with 1 mM ITPG at an OD_450_ of about 10 (1.86 g_cdw_ L^-1^) to obtain sufficient cell densities. After induction, the biomass concentration stayed almost constant during the rest of the experiment. By overexpressing the 2-KIV and the Ehrlich pathway, up to 13 mM isobutyric acid was produced in both strains. The wild type degraded the acid over the whole course of the experiment, traceable with the formation of 3-hydroxyisobutyric acid, a degradation product of isobutyric acid. The degradation was pronounced by glucose depletion resulting in the complete product metabolization within the next 30 h. In mutant C18 T7 no isobutyric acid degradation and, connected to that no 3-hydroxyisobutyric acid formation, was detectable. In both cases, glucose is partially converted to gluconate prior to the utilization for product or biomass generation. While the wild type completely exploited the carbon sources, C18 T7 did not completely convert the generated gluconate.

### Deletion of pathways competing for 2-ketoisovalerate and pyruvate increased carbon yield

**Figure 5:**
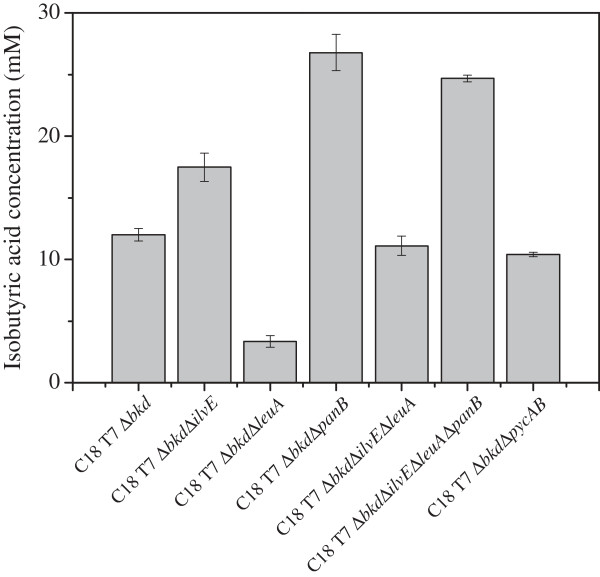
**Fermentation of different C18 T7 mutant strains harboring the production plasmids pPAPC-km and pPDPK.** Cultivations were done in shake flasks in M9* pH 7.4 starting with 20 g L^-1^ glucose and 5 g L^-1^ yeast extract. Induction was conducted with 1 mM IPTG at OD_450_ of 10. Error bars indicate standard deviations (n = 2).

In addition to the direct bypass of 2-KIV in the valine degradation pathway (catalyzed by *bkd*), 2-KIV is the precursor for the amino acids valine and leucine and the vitamin pantothenic acid in Pseudomonas. The branched-chain amino acid aminotransferase IlvE catalyzes, amongst others, the transamination of 2-KIV to valine. The 2-isopropylmalate synthase LeuA catalyzes the first step in leucine synthesis and PanB is a 2-ketoisovalerate hydroxymethyltransferase. To optimize the carbon yield, these competing pathways for 2-KIV were deleted. In addition, the genes coding for the pyruvate carboxylase (*pycAB*) were removed to prevent pyruvate withdrawal into the TCA-cycle due to anaplerosis. Single knockout mutants of Δ*ilvE,* Δ*leuA,* Δ*panB*, and Δ*pycAB*, and combined mutants of genes involved in 2-KIV metabolism were created and strains were transformed with production plasmids pPAPC-km and pPDPK. Figure 5 shows the results of fermentations conducted with a starting concentration of 20 g L^-1^ glucose in shake flasks. By deleting *panB*, the titer could be increased about 2.5-fold, *ilvE* deletion lead to a 1.5-fold increase. Mutants without a functional 2-isopropylmalate synthase showed decreased titers in single- as well as in double knock-outs. The deletion of the *pycAB* genes did not significantly influence the final titer of isobutyric acid.

## Discussion

In the present study, different variants of recombinant *Ps.* sp. strain VLB120 have been constructed by means of directed and random mutagenesis, which are capable of producing significant amounts of C4-precursors from glucose in aerobic fermentations with the focus on isobutyric acid synthesis.

### Isobutyraldehyde metabolism in *Ps.* sp. strain VLB120

Overexpressing simply one gene encoding for a branched chain 2-ketoacid decarboxylase (KivD) in *Ps*. sp. strain VLB120, enabled this organism to produce isobutyric acid, isobutanol, and 3-methyl-1-butanol, directly from glucose. Isobutyraldehyde was not detected in the fermentation broth, indicating direct intracellular conversion of this compound. Based on genome analysis, 32 aldehyde dehydrogenases (Additional file 1: Table S2), and about 19 alcohol dehydrogenases (Additional file 1: Table S3) have been identified in *Ps.* sp. strain VLB120 capable to either reduce or oxidize isobutyraldehyde. Among these enzymes are ALDHs homologs known to be highly active towards isobutyraldehyde (e.g. PVLB12825, a 42% homolog to *padA*[7] of *E .coli* K12). In addition, the strain harbors ADHs known to be able to catalyze the reduction of isobutyraldehyde (e.g. PVLB10545, a 86% homolog to *adhP*[41] of *E. coli* K12). However, gene deletions of these candidate genes did not significantly improve product titers (data not shown). Rodriguez et al. [41], aiming to overproduce isobutyraldehyde in *E. coli* BW25113, identified eight ADHs in the genome of *E. coli* BW25113 and observed that the final product titer could only be increased by combining multiple ADH deletions. While in *E. coli* BW25113 the degradation of isobutyric acid is not described [7], the present work shows that *Ps.* sp. strain VLB120 harbors a complete pathway for isobutyric acid utilization (Figure 1), which enabled *Ps.* sp. strain VLB120 to grow on isobutanol, isobutyraldehyde and isobutyric acid (Table 2). The existence of an isobutyric acid degradation pathway was already described for *P. putida*, *Candida rugosa*, *Yarrowia lipolytica* and *Desulfococcus multivorans* and used in mutant strains of the first three species for the synthesis of 3-hydroxyisobutyric acid using isobutyric acid as precursor [36,42-45].

### NTG-random mutagenesis to prevent isobutyric acid degradation

To prevent undesired isobutyric acid degradation, several mutants were created by random mutagenesis. The apparent correlation between isobutyric acid and 3-hydroxyisobutyric acid accumulation (in mutants B57, B83 and D67) indicates the *mmsAB* operon (PVLB_03765, PVLB_03770) [46] coding for methylmalonate-semialdehyde-dehydrogenase and 3–hydroxyisobutyrate dehydrogenase to be involved in the reaction of isobutyric acid to isobutyryl-CoA. Exemplarily, the respective DNA loci were sequenced in mutants B83 and C18. The DNA sequencing revealed, that *mmsB* is altered in variant B83. A transition (G/C → A/T) of nucleotide 413 occurred, confirming that this special transition is favored using NTG mutagenesis [47]. On the protein level, this mutation leads to an exchange of the strictly conserved residue Gly137 with asparagine [48] and thereby explains the non-active 3-hydroxyisobutyirc acid dehydrogenase in mutant B83. Mutant C18 shows no alteration in the *mmsAB* operon. In this case, most probably a broad substrate range acetyl-CoA-synthetase or a global regulator involved in isobutyric acid degradation was affected by NTG mutagenesis [45,49].

### Fermentative production of isobutyric acid in engineered *Ps.* sp. strain VLB120

The efficient fermentative production of isobutyric acid requires the overexpression of the genes of the valine synthesis pathway (*alsS*, *ilvC*, *ilvD*), a keto acid-decarboxylase (*kivd*) and a highly expressed gene for an aldehyde dehydrogenase (ALDH). While *ilvC*, *ilvD* and the ALDH gene are expressed homologously, *alsS* and *kivd* are derived from other species and show an altered codon usage, which often results in lower expression levels [50]. In order to maximize expression levels and overcome promoter related problems like catabolite repression by various carbon sources [51], the so far used P*alk* promoter was replaced by a T7 RNA polymerase based system. Using eGFP fluorescence as a fast and easily detectable read-out for expression (Additional file 1: Figure S2), the performance of this system was evaluated confirming recently published results for *P. putida* KT2440 T7 [52]. In addition, enzyme activity assays of single pathway genes proved successful overexpression in *Ps.* sp. strain VLB120 T7 (Table 3).

Overexpression of the 2-KIV synthesis pathway genes and *kivd* in the T7 variants of *Ps.* sp. strain VLB120 resulted in drastically increased isobutyric acid titers (Figure 4/ Table 1). In addition, the variant C18 T7 showed an increased stability of the formed product, which confirms the results of the 2-KIV biotransformations (Figure 2) under fermentative conditions. During glucose utilization, gluconate accumulated in *Ps.* sp. strain VLB120. This phenomenon is well understood and described in *Pseudomonas* species, which are known to metabolize glucose exclusively via the Entner-Doudoroff pathway, using 6-phosphogluconate as key intermediate [53]. *Ps.* sp. strain VLB120 possesses a glucose dehydrogenase (PVLB_05240), but is lacking a gluconate dehydrogenase, which prevents 2-ketogluconate formation. The incomplete conversion of gluconate in mutant strain C18 T7 is comparable to the behavior of *Pseudomonas putida* KT2440 during poly-hydroxyalkonate synthesis observed by Poblete-Castro et al. [54]. In their work, they enhanced the production of PHAs by the deletion of glucose dehydrogenase without affecting the specific growth rate. One may speculate, that this deletion leads to an increased flux towards pyruvate and thus more precursors for isobutyric acid synthesis are available.

### Optimization of isobutyric acid production by the deletion of competing pathways

To prevent unproductive 2-KIV depletion via isobutyryl-CoA or various amino acid synthesis pathways (Figure 1), the influence of the genes *bkd*, *ilvE*, *leuA, pycAB* and *panB* was investigated. The strongest effect on isobutyric acid titers was measured for the mutants Δ*panB* and Δ*bkd.* By deleting subunit A of the branched chain α-keto acid dehydrogenase complex (*bkd*), growth on 2-KIV could be completely inhibited in *Ps.* sp. strain VLB120. Growth could be restored by overexpressing the decarboxylase gene *kivd*, channeling 2-KIV over isobutyraldehyde (Figure 1). The mutant strain shows drastically increased product concentrations of isobutyric acid and isobutanol during 2-KIV biotransformations, while during growth on glucose (fermentation) this effect seems to be insignificant, as the production rate of *Ps*. sp VLB120Δ*bkd* C18-T7 harboring Δ*bkd* is comparable to *Ps.* sp VLB120*.* Under fermentative conditions comprising high glucose und only low 2-KIV concentrations the activation of 2-KIV to isobutyryl-CoA seems to be only a minor carbon sink. Similar observations have been reported by Lu et al. [55] for *Ralstonia eutropha.* Deletion of *ilvE* helped to prevent undesired carbon loss, this confirms similar results reported for *C. glutamicum* and *R. eutropha*[55-57].

Deletion of *panB* had the strongest impact on carbon yield for isobutyric acid synthesis. *panB* is the first gene of pantothenic acid synthesis pathway, and in addition a precursor for CoA [58]. The deletion of *panB* seems to increase 2-KIV availability, as also described for *C. glutamicum*[59,60], and thereby results in an increased carbon yield of 0.12 ± 0.01 g g^-1^, which is about 25% of the theoretical maximum. *panB* deletion leads to mutants unable to grow on minimal medium (most probably connected to the disability to synthesize CoA).

For *E. coli* BW25113 10 g L^-1^ on a 5 mL scale using 40 g L^-1^ glucose has been reported [7] while for *Ps.* sp. strain VLB120 2 g L^-1^ have been observed using only 20 g L^-1^ carbon source. Experiments have been conducted in shake flasks with glucose excess where gluconate temporarily accumulated resulting in a pH drop to pH 6.8 which was even more pronounced during isobutyric acid formation. To prevent this pH drop, hampering catalyst robustness, glucose needs to be limited and the pH controlled. It is to be expected, that in such a controlled environment (bioreactor) higher final product titers and better carbon yields will be reached.

### Isobutyric acid production and beyond

In consequence, the application of the demonstrated design principles, the use of the genome sequence of *Ps*. sp. strain VLB120 and the established metabolic engineering tools, will give access to a far broader product spectrum. Based on the engineered, highly active 2-KIV synthesis pathway the production of isobutanol, 3-methyl-1-butanol, 3-hydroxyisobutyric acid, isobutyraldehyde, valine and D-pantothenate would be feasible by slight pathway modifications. Beside the synthesis of isobutyric acid, we were already able to detect the Ehrlich pathway products isobutanol and 3-methyl-1-butanol, which are interesting bulk chemicals [33,61]. More over 3-hydroxyisobutytric acid was accumulated in the not optimized mutant B83 with remarkable titers, being highly valuable synthons for the fine chemical industry [62].

## Conclusion

The combination of an increased flux towards isobutyric acid using a tailor-made expression system and the prevention of precursor and product degradation allowed efficient production of isobutyric acid in *Ps*. sp. strain VLB120. This work experimentally verifies the genome derived metabolic network structure. A true platform organism was designed, which has the ability to produce an even wider product spectrum directly from glucose by only slight changes on the level of cell metabolism.

In general, studies investigating *Pseudomonas* for the fermentative production of chemicals are few [19]. But clear benefits like low/no byproduct formation during glucose fermentation, high tolerance towards toxic substances [12], improved NAD(P)H regeneration rates under stress conditions [21], and a diverse gene repertoire for the processing of organic molecules underline the potential of *Pseudomonads* for industrial applications. The strain engineering of this novel organism sets the stage for the development of aerobic biofilm based processes for the continuous production of isobutyric acid and other secondary metabolites. Apart from increasing product titers, the long-term catalyst robustness is a most important but often neglected issue in host engineering, which will be a key focus in future studies regarding this newly introduced organism.

## Methods

### Chemicals, bacterial strains, and plasmids

All chemicals used in this study were purchased from Sigma-Aldrich (Steinheim, Germany) or Carl Roth GmbH (Karlsruhe, Germany), unless stated otherwise. The chemicals were of the highest purity available and used without further purification. The bacterial strains and plasmids used in this study are listed in Table 4.

**Table 4:** Bacterial strains and plasmids used in this study

**Strains**	**Relevant characteristic(s)**	**Reference**
*E. coli* DH5α	*supE44,* D*lacU169 (*f*80 lacZ*D*M15), hsdR17 (rk-mk*+*), recA1, endA1, thi1, gyrA, relA*	[63]
*E. coli* DH5α ʎ-pir	ʎ-*pir* lysogen of DH5a	[64]
*Ps.* sp. strain VLB120	Wildtype strain	[65]
*Ps.* sp. strain VLB120 T7	P_lacUV5_ T7 RNA pol, *lacI*^q^	This study
*Ps.* sp. strain VLB120Δ*bkd*	VLB120 with deletion of *bkd*, encoding subunit A of the branched-chain α-keto acid dehydrogenase	This study
*Ps.* sp. strain VLB120Δ*bkd* A10, A21, B57, B83, C18, D67, D76, E82	NTG-mutants of *Pseudomonas* sp. strain VLB120 Δ*bkd*	This study
*Ps.* sp. strain VLB120Δ*bkd* C18 T7	P_lacUV5_ T7 RNA pol, *lacI*^q^	This study
*Ps.* sp. strain VLB120Δ*bkd*Δ*ilvE* C18 T7	*Ps.* sp. strain VLB120Δ*bkd* C18 Δ*ilvE*, encoding branched-chain amino acid aminotransferase	This study
*Ps.* sp. strain VLB120Δ*bkd*Δ*pycAB* C18 T7	*Ps.* sp. strain VLB120Δ*bkd* C18 Δ*pycAB*, encoding pyruvate decarboxylase	This study
*Ps.* sp. strain VLB120Δ*bkd*Δ*leuA* C18 T7	*Ps.* sp. strain VLB120Δ*bkd* C18 Δ*leuA*, encoding 2-isopropylmalate synthase	This study
*Ps.* sp. strain VLB120Δ*bkd*Δ*panB* C18 T7	*Ps.* sp. strain VLB120Δ*bkd* C18 Δ*panB*, encoding 2-ketoisovalerate hydroxymethyltransferase	This study
*Ps.* sp. strain VLB120ΔbkdΔ*ilvE*Δ*panB* C18 T7	*Ps.* sp. strain VLB120Δ*bkd*Δ*ilvE* C18 Δ*panB*, encoding 2-ketoisovalerate hydroxymethyltransferase	This study
*Ps.* sp. strain VLB120Δ*bkd*Δ*ilvE*Δ*leuA* C18 T7	*Ps.* sp. strain VLB120Δ*bkd*Δ*ilvE* C18 Δ*leuA*, encoding 2-isopropylmalate synthase	This study
*Ps.* sp. strain VLB120Δ*bkd*Δ*ilvE*Δ*leuA*Δ*panB* C18 T7	*Ps.* sp. strain VLB120Δ*bkd*Δ*ilvE*Δ*leuA* C18 T7Δ*panB*, encoding 2-ketoisovalerate hydroxymethyltransferase	This study
**Plasmids**	**Relevant characteristic(s)**	**Reference**
pMA-RQ-*kivd*	Amp^r^*,* Geneart delivery vector containing *kivd*	This study/Geneart
pCOM10	Km^r^ , broad host range expression vector, *alk* promoter, *oriT*, *alkS* regulator gene,	[66]
pCOM10-*kivd*	pCOM10 containing *kivd*	This study
pCOM10-*eGFP*	pCOM10 containing *eGFP*	This study
pBR22b	Cm^r^, mob *lacI*^*q*^*Km*^*r*^ PT7Ф10	[67]
pBR22b-*kivd*	pBR22b containing *kivd*	This study
pBR22b-*alsS*	pBR22b containing *alsS*	This study
pBR22b-*ilvC*	pBR22b containing *ilvC*	This study
pBR22b-*ilvD*	pBR22b containing *ilvD*	This study
pBR22b-*alsS-*km	Cm^r^, Km^r^, pBR22b-*alsS* with BoxI blunt end inserted Km^r^	This study
pPAPC-km	pBR22b-alsS-km additionally containing *ilvC*	This study
pBR22b-*eGFP*	pCOM10 containing *eGFP*	This study
pBR22b-*eGFP-*km	Cm^r^, Km^r^ pBR22b-eGFP with BoxI blunt end inserted Km^r^	This study
pCOM8	Gm^r^, broad-host-range expression vector, *alk* promoter, *oriT*, *alkS* regulator gene	[66]
pCOM8-T7-*ilvD*	pCOM8 containing *ilvD*	This study
pPDPK	pCOM8-T7-*ilvD* containing *kivd*	This study
pJQ200SK	Gm^r^, suicide vector, P15A *ori sac*B RP4 pBluescriptSK MCS	[68]
PJQ200SK-*bkd*-KO	Knock-out vector	This study
pJQ*hdp*::km	Km^r^, Gm^r^, pJQ200SK with *hdp* disrupted by Km^r^ gene, flanked by *loxP* recombination sites	Nick Wierckx (unpublished data)
pJTN-*cre*	*Gm*^*r*^, encodes *cre* recombinase	Nick Wierckx (unpublished data)
pUC18 mini-Tn7-Gm-T7	pUC18, mini-Tn7-Gm, P_lacUV5_ T7 RNA pol, *lacI*^q^	[52]
pUC18 mini-Tn7-Gm-T7	pUC18, mini-Tn7-Gm, P_lacUV5_ T7 RNA pol, *lacI*^q^	[52]
pTNS1	Amp^r^, *TnsABCD*	[69]
pEMG	Km^r^, oriR6K, *lacZa* with two flanking I-SceI sites	[64]
pSW-2	Gm^r^, *ori*RK2, *xylS*, Pm→ *I-SceI*	[64]
pEMG-*pycAB*-KO	Knock-out vector	This study
pEMG-*ilvE*-KO	Knock-out vector	This study
pEMG-*leuA*-KO	Knock-out vector	This study
pEMG-*panB*-KO	Knock-out vector	This study

Amp^r^: ampicillin resistance; Cm^r^: chloramphenicol resistance; Km^r^: kanamycin resistance; Gm^r^: gentamicin resistance.

### Cultivation of bacterial strains

*Ps.* sp. strain VLB120 was used as production host, while *E. coli* DH5α and *E. coli* DH5α (λ-pir) served as host strains for all plasmid based DNA manipulations. *Ps.* sp. strain VLB120 and *E. coli* strains were cultivated at 200 rpm, 30°C or 37°C (Infors AG, Bottmingen, Switzerland), respectively. For all cloning purposes, transformations and first precultures, cells were cultivated in lysogeny broth (LB, [70]). All other cultivations were performed in M9* minimal medium [71] which was supplemented with 1 mL L^-1^ US* trace element solution [72] and 2 mL L^-1^ 1 M MgSO_4_ solution. For fermentations and cultivations used to prepare cell extracts, 5 g L^-1^ yeast extract and 0.001% (w/v) thiamine solution was added. Depending on the experiment, either glucose (5 g L^-1^ or 20 g L^-1^), isobutyric acid (10 mM), isobutyraldehyde (10 mM), isobutanol (10 mM), succinate (10 mM) or 2-ketoisovalerate (10 mM) served as carbon sources. All media were supplemented with appropriate antibiotics (kanamycin 50 μg mL^-1^ for *E. coli* and *Ps.* sp. strain VLB120, gentamycin 25 μg mL^-1^ for *E. coli* and *Ps.* sp. strain VLB120, chloramphenicol 34 μg mL^-1^ for *E. coli*)*.*

For cultivations in minimal media, *Ps.* sp. strain VLB120 was cultivated in two subsequent precultures prior to the main culture. First, the strain was cultivated for 8 h in tubes filled with 5 mL LB overnight. 50 μL of this culture was used as inoculum for a fresh 5 mL M9* culture. After overnight growth 50 mL M9* medium in a 250 mL flask were inoculated to an initial OD_450_ 0.2. *Ps.* sp. strain VLB120 cultures were either grown under aerobic conditions in open baffled shake flasks or under micro aerobic conditions in sealed baffled shake flasks to prevent the evaporation of volatile compounds.

For experiments investigating the utilization of different carbon sources by *Ps.* sp. strain VLB120 preculture derived cells were washed with M9* salt solution before inoculating fresh M9* medium complemented with the respective carbon source.

### Random mutagenesis by N-methyl-N’-nitro-N-nitrosoguanidine (NTG)

Random mutagenesis by NTG was performed according to Adelberg et al. and Martin et al. [37,40]. 5 mL M9*-medium (pH 7.4, 10 mM succinate as carbon source) was inoculated with 50 μL of an 8 h LB culture of *Ps. sp.* strain VLB120Δ*bkd* and cultured overnight. This culture was used to inoculate the main culture (250 mL M9*, 10 mM succinate). Early exponential phase cells were harvested by centrifugation (10 min, 4.500 *g*, 4°C), washed and concentrated in CpI-Buffer (127 mM Na_2_HPO_4_∙2H_2_O, 36.2 mM Citric acid, pH 6.0) to an OD_450_ of 6 before 80 μg mL^-1^ NTG was added. Cells were incubated for 30 min, washed, resuspended in LB and stored for screening in 10% (w/v) glycerol at −80°C.

### Screening for NTG-mutants unable to degrade isobutyric acid

To screen for mutants with changed properties regarding isobutyric acid degradation, 5 mL M9* medium containing 10 mM succinate were inoculated with NTG treated cells (see above). After overnight cultivation, cells were harvested by centrifugation, washed in identical medium without carbon source, and used to inoculate fresh 5 mL M9* medium with 10 mM isobutyric acid as sole carbon source to an OD_450_ of 0.2. After reaching an OD_450_ of 0.4, solid ampicillin was added up to its solubility limit (13 mg mL^-1^) and cultivation was continued for additional 8 hours. Afterwards, cells were harvested by centrifugation, washed twice with M9* medium, plated in appropriate dilutions to M9* plates containing 10 mM succinate as carbon source, and incubated at 30°C for 48 h. Mutants were replica plated on M9* medium containing 10 mM isobutyric acid and on LB medium. Mutants unable to grow in the presence of isobutyric acid as sole carbon source were selected for further investigations.

### Enzyme assays

For the preparation of cell extracts, cells were cultivated in M9*(pH 7.4, 20 g L^-1^ glucose) and depending on the plasmid used induced either with 1 mM IPTG or 0.05% DCPK during exponential phase (OD_450_ 0.5). After 4 h of induction, cells were harvested by centrifugation, washed, and resuspended to cell density of OD_450_ 100 in ice cold buffer (50 mM Kpi pH 7.0, 0.5 mM dithiothreitol). Cells were disrupted by three passages through a precooled French press (5.5 MPa, SLM-Aminco, Rochester, NY, USA) and cellular debris was removed by centrifugation (20 min, 17,000 *g*, 4°C). Cell extracts were always prepared freshly and kept on ice. Total protein concentrations were determined using the Bradford protein assay [71].

The activity of acetolactate synthase (AlsS) was assayed according to Lang et al. [73], using 50 mM Kpi buffer (pH 7.0). The activity of ketol-acid reductoisomerase (IlvC) was determined according to Leyval et al. [74]. The assay was coupled to the activity of AlsS using 50 mM pyruvate as substrate instead of 2-acetolactate. The activity of dihydroxy acid dehydratase (IlvD) was quantified according to Atsumi et al. [75]. The substrate, 2,3-dihydroxy-isovalerate was purchased from SelectLab Chemicals GmbH (Bönen, Germany). The assay was conducted at 30°C for 30 min. 2-keto acid decarboxylase (Kivd) activity was determined by measuring the isobutyraldehyde concentration using HPLC analysis. Therefore, 500 μL 50 mM potassium phosphate buffer (pH 7.0) containing 10 mM MgCl_2_, 0.5 mM thiamine diphosphate, cell free extract, and 10 mM 2-KIV was incubated at 30°C for 30 min. The reaction was started by the addition of 2-KIV and stopped by the addition of 10 μL pure perchloric acid.

### Analytical methods

Cell densities were monitored by measuring the optical density at 450 nm (OD_450_) using a spectrophotometer (Biochrom Libra S12, Cambridge, UK). One OD_450_ unit corresponds to 0.186 g_cdw_ L^-1^ for *Ps.* sp. strain VLB120.

Organic acids, sugars, alcohols and aldehydes were detected by HPLC (LaChrom Elite, Merck Hitachi, Darmstadt, Germany) equipped with a Trentec 308R-Gel.H ion exclusion column (300 × 8 mm, Trentec Analysentechnik, Gerlingen, Germany). The following conditions were applied; temperature: 40°C, isocratic flow rate: 1.0 ml min^-1^, solvent: 5 mM H_2_SO_4_, injection volume: 20 μL. Analytes were detected either by a UV (λ = 210 nm) or RI detector.

Lower product concentrations (< 500 μM analyte) were quantified on a Finningan Focus GC (Thermo Electron Corporation, Dreireich, Germany) with a Rt-βDex-sm column (30 m × 0.25 mm × 0.25 μm, Restek GmbH, Bad Homburg, Germany): GC oven temperature was 40°C for 5 min before it was increased to 80°C at a rate of 20°C min^-1^, and to 105°C at a rate of 7°C min^-1^. The maximum temperature of 200°C was reached at a rate of 60°C min^-1^ and kept for 3 min. Samples were extracted with 1:1 (v/v) ice cold diethyl ether containing 0.2 mM dodecane as internal standard.

eGFP fluorescence was measured using a microtiter plate reader (Infinite M200, Tecan, Mannedorf, Switzerland) at 488 nm and 511 nm for excitation and emission, respectively. Cells were harvested by centrifugation, washed and resuspended in 50 mM Tris buffer pH 8.0. Biomass concentrations were adjusted to OD_450_ 0.4 using 50 mM Tris-Cl pH 8.0.

### DNA manipulation

Restriction enzymes, T4 ligase, thermosensitive alkaline phosphatase, T4 polynucleotide kinase, and high-fidelity phusion DNA polymerase were purchased from Fermentas GmbH (St. Leo-Rot, Germany). All enzymes were used according to supplier’s recommendations. Plasmid DNA was isolated with a miniprep Kit (Peqlab GmbH, Erlangen, Germany). Gel extraction and DNA purification were performed with a NucleoSpin Gel and PCR clean-up Kit (Macherey-Nagel GmbH, Düren, Germany). All DNA manipulations were performed using standard procedures [71]. Restriction sites, which were added to the respective DNA fragments using PCR amplification, are listed in the primer section (Additional file 1: Table S2).

Transformations were performed by electroporation (2500 V, Equibio EasyjecT Prima, Ashford, UK) using sucrose-treated competent cells [76] and glycerol-treated competent cells [71] for *Ps.* sp. strain VLB120 and *E. coli* DH5α, respectively.

#### pCOM-based plasmid construction

The *kivd* gene was artificially synthesized (Geneart AG, Regensburg, Germany) in pMA-RQ-*kivd* based on available sequence data of *Lactococcus lactis* subsp. lactis strain IFPL730 (GenBank: AJ746364). *kivd* was cut from the original delivered plasmid at artificially inserted restriction sites NdeI and AscI and ligated into pCOM10 creating pCOM10-*kivd. eGFP* was amplified using primers KL35/36 digested and ligated into pCOM10 to create pCOM10-*eGFP*.

#### Single gene pBR22b constructs

pCOM10-*kivd* was cut with NdeI and SalI and the fragment was ligated into pBR22b creating pBR22b-*kivd*.

The *alsS* gene was amplified in two fragments from genomic *Bacillus subtilis* DNA using primers KL1/2 and KL3/4, respectively, which were designed based on available sequence data (Genbank: AP012496 region: 3537181). The fragment was ligated into pBR22b creating pBR22b-*alsS*. To ensure an appropriate antibiotic selection pressure in *Ps. sp.* strain VLB120, a kanamycin cassette was amplified from pJQ*hdp*::Km by primers KL11/12 and the phosphorylated fragment was ligated into dephosphorylated pBR22b-*alsS* cut with BoxI, creating pBR22b-*alsS*-Km.

*ilvC* (PVLB_03705) and *ilvD* (PVLB_01425) were amplified from genomic *Ps. sp.* strain VLB120 DNA using primers KL 5/6 and KL 7/8, respectively. The fragments were inserted in pBR22b creating pBR22b-*ilvC* and pBR22b-*ilvD*.

*eGFP* was amplified using primer KL35/36 digested and ligated into pBR22b to create pBR22b-*eGFP*.

#### Construction of pPAPC-Km and pPDPK

*ilvC* including the T7 promoter and the ribosomal binding site (RBS) was amplified from pBR22b-*ilvC* with primers KL13/14. The fragments were inserted into the dephosphorylated vector pBR22b-*alsS*-Km creating pPAPC-Km.

*ilvD* including the T7 promoter, the RBS, and the T7 terminator was amplified from pBR22b-*ilvD* using primers KL29/30 and the phosphorylated, digested fragment was ligated into the dephosphorylated Bst1107I cut pCOM8 creating pCOM8-*ilvD*. Orientation of the fragment was determined using XhoI restriction and the vector with the same orientation of *ilvD* and Gm^r^ cassette was used for further work.

*kivd* including the T7 promoter, and the RBS was amplified from pBR22b-*kivd* using primers KL27/28 and the phosphorylated, cut fragment was ligated into the dephosphorylated, restricted pCOM8-*ilvD* creating pPDPK.

#### Gene deletion (Δbkd)

Deletion of the gene coding for subunit A of the branched chain α-keto acid dehydrogenase (*bkd*) was achieved as described earlier using pJQ200SK [68]. For the construction of pJQ200*bkd*::Km, genomic *Ps.* sp. strain VLB120 DNA was used as template to amplify the up- and downstream fragment of *bkd* using primers K15/16 and K17/18. Km^r^-loxP was cut from pJQ*hdp*::Km with XbaI and purified by gel electrophoresis. All three parts were ligated in one pot and *Ps.* sp. strain VLB120 was transformed with the verified vector. Kanamycin resistance was used to prove genomic integration of pJQ200*bkd*::Km. To distinguish between single crossover and double crossover events, mutants were screened by replica plating for kanamycin resistance and gentamycin sensitivity. Positive clones were confirmed using colony PCR with primers KL15/18. Km^r^-cassette was removed by using *cre* recombinase. Positive mutants were transformed with pTnCre and a single colony was picked to inoculate a 5 mL LB culture including 0.1 mM sodium salicylate to induce *cre* recombinase expression. After 4 h, the culture was appropriately diluted and resulting kanamycin sensitive colonies were cured from pTNCre by subsequent inoculation and screening for gentamycin sensitive colonies.

#### Gene deletions (ΔilvE, ΔleuA, ΔpanB, ΔpycAB)

To obtain all other deletion mutants, the recently published gene editing method for *Pseudomonas* species using the pEMG/pSW-2 system was used [64]. This system allows faster identification of positive clones compared to the pJQ200SK method. Up- and downstream regions of the desired genes were amplified by PCR using the following primers (KL19/20 and KL21/22 for *ilvE*, KL23/24 and KL25/26 for *pycAB*, KL37/38 and KL39/40 for *leuA,* KL41/42 and KL43/44 for *panB*) and purified via gel electrophoresis. The resulting up- and downstream fragments were fused by PCR using primers (KL19/22 for *ilvE*, KL23/26 for *pycAB*, KL37/40 for *leuA*, KL41/44 for *panB*) and after gel electrophoresis, cut and ligated into the identically treated pEMG vectors. *Ps.* sp. strain VLB120 was transformed with the respective plasmids and single crossover mutants were identified using cPCR. Positive clones were transformed with pSW-2. *I-SceI* nuclease activity was already present in *Ps.* sp. strain VLB120 pSW-2 without additional induction of *xylS* with 3-methylbenzoate, thus colonies were replica plated to screen for kanamycin sensitive mutants. Gene deletion mutants were identified by cPCR using primers KL31/32 for *ilvE*, KL33/34 for *pycAB,* KL45/46 for *leuA*, and KL47/48 for *panB*. Positive clones were cured from the pSW-2 plasmid by repeated inoculation and screening for gentamycin sensitive mutants.

#### Construction of Pseudomonas sp. strain VLB120 T7

To establish *Ps.* sp. strain VLB120 as host for aerobic fermentations, the gene encoding for a T7 RNA polymerase under the control of a UV5 promoter and its regulator gene *lac*I^q^ were integrated in the genome using a Tn7-transposon. Km^r^-loxP*,* was isolated from pJQhdp::Km with XbaI, purified by gel electrophoresis and ligated into XbaI cut, dephosporylated mini-Tn7-T7-Gm creating mini-Tn7-T7-KmLoxP. *Ps.* sp. strain VLB120 was transformed by electroporation with mini-Tn7-T7-KmLoxP and pTNS1 and kanamycin resistant mutants were checked by cPCR and primers KL9/10 for correct genomic integration of genes coding for *lacI*^q^ and T7 RNA polymerase. The Km^r^-cassette was removed as described above. To compare the effectiveness of the two different expression systems, eGFP fluorescence was used as a fast and easily detectable read-out for expression (Additional file 1: Figure S2). Depending on the carbon source, the fluorescence signal is about 3–6 fold higher in pBR22b-*eGFP* carrying strains, as compared to strains carrying pCOM10-*eGFP*. In both systems, the highest eGFP levels could be obtained on citrate as carbon source whereas in the presence of glucose only half of the fluorescence signal could be measured. The biggest difference was observed in complex medium; an approximately 6-fold higher eGFP concentration could be detected in *Ps.* sp. strain VLB120 pBR22b-*eGFP*. With regard to the fermentative production of isobutyric acid, the utilization of complex medium compounds might increase the maximum achievable product concentrations (Table 1[61]). In addition, the *eGFP* expression for pBR22b as well as for pCOM10 was found to be tightly controlled in *Ps.* sp. strain VLB120 (T7). Without induction only a negligible amount of *eGFP* expression was detected.

## Competing interests

The authors declare that they have no competing interests.

## Authors’ contributions

KL and KB designed experiments; KL and JZ performed the experiments; KL, KB and AS analyzed the data; KL and KB wrote the paper. All authors read and approved the final manuscript.

## Supplementary Material

Additional file 1 **Oligonucleotides used in this study (Table S1), lists of putative aldehydeand alcohol dehydrogenases of Pseudomonas sp. strain VL120 (Table S2+3) and supplemental experimental data (Figure S1-5).** Click here for file
