# Supplementary material for: Metabolic engineering of Pseudomonas sp. strain VLB120 as platform biocatalyst for the production of isobutyric acid and other secondary metabolites
Source: Microb Cell Fact. 2014 Jan 7;13:2. doi: 10.1186/1475-2859-13-2 (PMC3897908; doi:10.1186/1475-2859-13-2)
Supplement: Additional file 1 — Oligonucleotides used in this study (Table S1), lists of putative aldehydeand alcohol dehydrogenases of Pseudomonas sp. strain VL120 (Table S2+3) and supplemental experimental data (Figure S1-5). [file 1475-2859-13-2-S1.pdf]

Additional file 1

Table S. 1

Oligonucleotides used in this study

Oligonucleotide	Sequence	Description
KL1	GCGCCATATGTTGACAAAAGCAACAAAAGAAC	Amplification of *alsS* gene, NdeI
KL2	ATGCTCTAGAAAGGGTACCGGCAG	Amplification of *alsS* gene, XbaI
KL3	GCGCTCTAGAGATTTAGAGGATCAATATTTTGG	Amplification of *alsS* gene, XbaI
KL4	ATGCGGATCCCTAGAGAGCTTTCGTTTTCA	Amplification of *alsS* gene, BamHI
KL5	GCATCATATGAAAGTTTTCTACGACAA	Amplification of *ilvC* gene, NdeI
KL6	GCATAAGCTTTTAGTTCTTGGTCTTGTCGA	Amplification of *ilvC* gene, HindIII
KL7	GCATCATATGCCTGATTATCGTTCCAA	Amplification of *ilvD* gene, NdeI
KL8	GCATAAGCTTTTACAGCCCTTCGAGCATCG	Amplification of *ilvD* gene, HindIII
KL9	GGCGTTGGTAGACAGCGATATG	Primer to verify Tn7-transposon intergration
KL10	CCCTTTCAAGCTCAAGCCTCTC	Primer to verify Tn7-transposon intergration
KL11	ATATGACCCGTGTCCGCTGAGGTGGT	Amplification of Km^r gene
KL12	GCATGACCCGTGTCAAAGCCACG	Amplification of Km^r gene
KL13	CGATCCGTACGTAATACGACTCACTATAGGG	Amplification of *ilvC* gene, T7-promoter, RBS, BsiWI
KL14	CGATCCGTACGCCTAGGTTAGTTCTTGGTCTTGTCGA	Amplification of *ilvC* gene, BsiWI
KL15	ATATGCGGCCGCTGCGTTTGCATGTGCCCGAG	Primer for deletion of the *bkd* gene, up primer, NotI
KL16	GCATTCTAGAGCCAACGGCCTGGACGAACT	Primer for deletion of the *bkd* gene, up primer Xbai
KL17	GCGCTCTAGAATCAAGGGCGACACCAAGAT	Primer for deletion of the *bkd* gene, down primer Xbai
KL18	GCATGGATCCAGGTGATCGGGCATCTCCT	Primer for deletion of the *bkd* gene, down primer, Bamhi
KL19	GGCCGAGCTCCAAACGTGAACTGGCGCGTATGGAG	Primer for deletion of the *ilvE* gene, SacI
KL20	GGCTATCAGGCGACCTTGACGCTCTCGTTACTCATGAC ACCCTCG	Primer for deletion of the *ilvE* gene, fusion primer up
KL21	ATGCTCTAGAATCGGGCACCTGCGGTCACCCGACG	Primer for deletion of the *ilvE* gene, XbaI
KL22	GTGTCATGAGTAACGAGAGCGTCAAGGTCGCCTGATA GCCCACGC	Primer for deletion of the *ilvE* gene, fusion primer down
KL23	GTATCTCAGCCCTCGATCTCGATTTTTTTATCACTGGA GTCTTC	Primer for deletion of the *pyc* gene, fusion primer up
KL24	ATGCGAGCTCATCGCCAGCTTCAGCTGCCCCTGCA	Primer for deletion of the *pyc* gene, SacI
KL25	ATATGGATCCCGGACAGGCGCAGGGCATCCTGAGC	Primer for deletion of the *pyc* gene, BamHI
KL26	CTCCAGTGATAAAAAAATCGAGATCGAGGGCTGAGA TACGCCCT	Primer for deletion of the *pyc* gene, fusion primer down
KL27	GCGCGCGGCCGCTAATACGACTCACTATAGGGGAATT	Amplification of *kivd* gene, T7 promoter, NotI
KL28	ATATGCGGCCGCGGGCGCGCCTTATGATTTAT	Amplification of *kiv*d gene, NotI

KL29	CCTAGGTAATACGACTCACTATAGGGGAATTGTG	Amplification of *ilvD* gene, T7-promoter, RBS
KL30	GCGCGAGCTCTATAGTTCCTCCTTTCAGCA	Amplification of *ilvD* gene, T7-terminator
KL31	CAATGGCTCGGAGCAAGTC	Primer to verify deletion of the *ilvE* gene
KL32	CATCGTCGGCCATTCTGTG	Primer to verify deletion of the *ilvE* gene
KL33	GATCTTGCAGGCGCAAGTG	Primer to verify deletion of the *pyc* gene
KL34	CATCTACGCCTACGGCAGAAC	Primer to verify deletion of the *pyc* gene
KL35	GCCGCATATGGTTAGTAAGGGAGAGGA	Amplification of *eGFP* gene, NdeI
KL36	GCGCAAGCTTTTACTTGTACAGCTCGTCCA	Amplification of *eGFP* gene, HindIII
KL37	GCATGAGCTCGGCTACCGGGCCACATATGCACTGG	Primer for deletion of the *leuA* gene, SacI
KL38	AAGGCTCAGGCGGCCTTGGCTTTGAGCATGGTCATGGAAGCAATC	Primer for deletion of the *leuA* gene, fusion primer up
KL39	CTTCCATGACCATGCTCAAAGCCAAGGCCGCCTGAGCCTTACCCT	Primer for deletion of the *leuA* gene, fusion primer down
KL40	GCATTCTAGAGCGTGGCGGCAATCAAGGCCTTTGC	Primer for deletion of the *leuA* gene, XbaI
KL41	ATATGAGCTCGAAAATGACCAGGGGCGTGTGCGCA	Primer for deletion of the *panB* gene, SacI
KL42	TGTATTCATGCGCTGAACCCGGTTACTTCAGGCATTTAGGAAAAT	Primer for deletion of the *panB* gene, fusion primer up
KL43	CCTAAATGCCTGAAGTAACCGGGTTCAGCGCATGAATACAGTCAA	Primer for deletion of the *panB* gene, fusion primer down
KL44	GCATTCTAGACATGTTGAGGTCACGCACCATCGCC	Primer for deletion of the *panB* gene, XbaI
KL45	TCTGGTGGGCGACTACTTC	Primer to verify deletion of the *leuA* gene
KL46	CATCCAAGTGCTGCGTGAG	Primer to verify deletion of the *leuA* gene
KL47	GTGACGAGGCCTTCGATTG	Primer to verify deletion of the *panB* gene
KL48	CTTGGCCACGGGTAATCTG	Primer to verify deletion of the *panB* gene

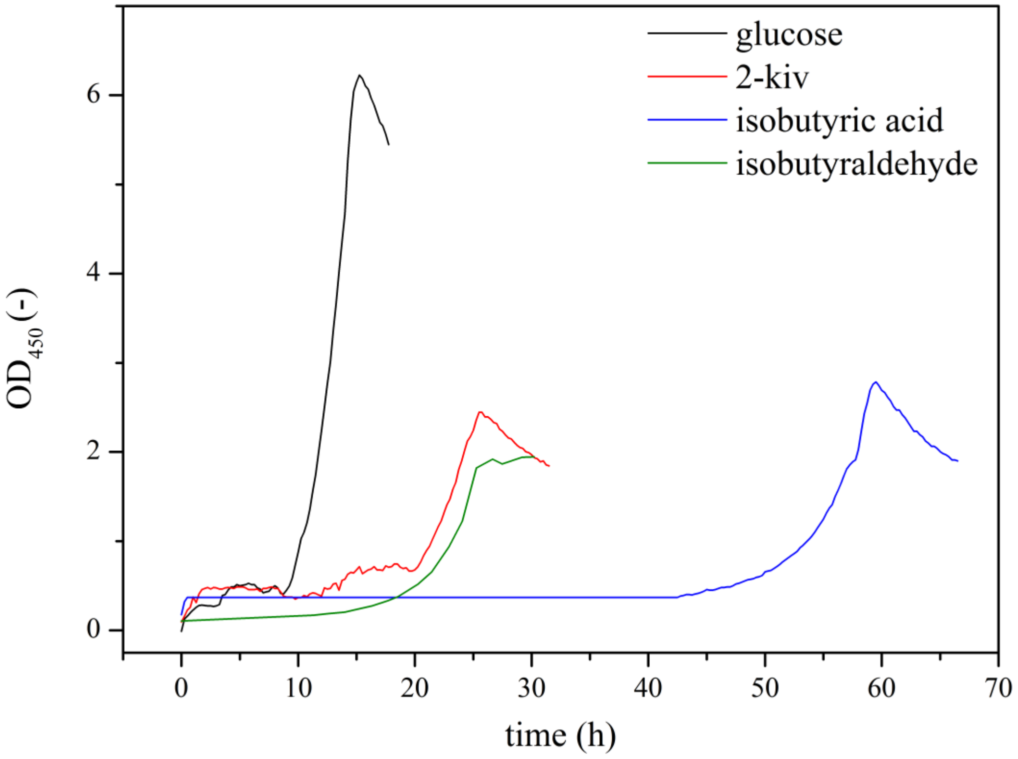

Figure S. 1. Growth behavior of *Pseudomonas* sp. strain VLB120 on different carbon sources. Cells were cultivated on 10 mM of different carbon sources in M9* medium pH 7.0. Experiments were performed 2-4 times.

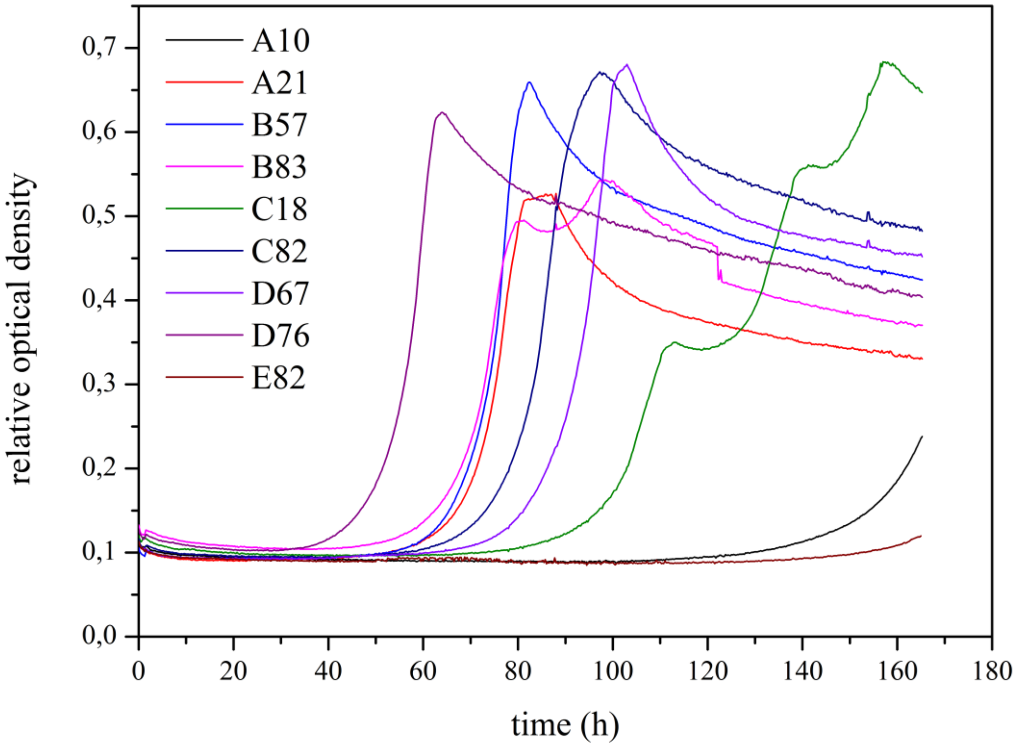

Figure S. 2. Growth behavior of NTG-mutant strains of *Pseudomonas* sp. strain VLB120Δbkd on M9* pH 7.0 with 10 mM isobutyric acid as sole carbon source. Experiments were performed 3 times.

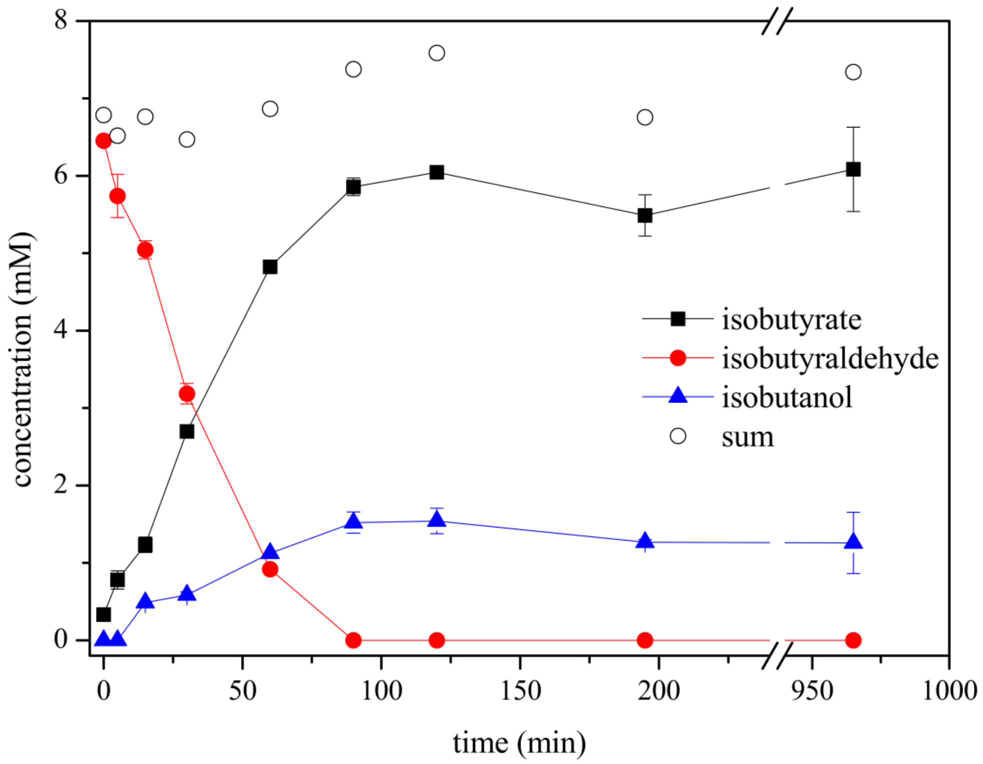

Figure S. 3. Resting cell biotransformation of *Pseudomonas* sp. strain VLB120 C18. Cells were cultivated in M9* pH 7.4 supplemented with 5 g L^{-1} of glucose. Assays were performed using 1 g_{cdw} L^{-1} of cells; reactions were started by adding 10 mM isobutyraldehyde; reactions were stopped by the addition of perchloric acid. Error bars indicate standard deviations (n = 2).

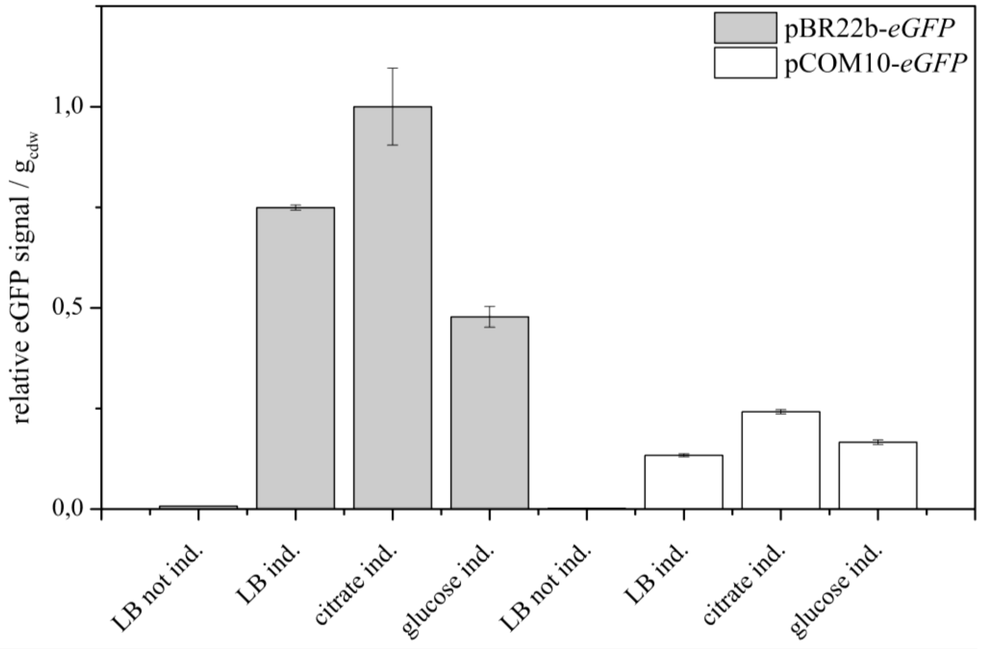

Figure S. 4. Comparison of the two different expression systems P_{alk} and P_{T7}. Promoter performance was deduced from the relative eGFP signal strength and values were normalized to the maximum using *P*. sp. strain VLB120 (T7) carrying either pBR22b-eGFP of pCOM10-eGFP. Growth medium was M9* pH7.4 with 0.5% of the respective carbon source, or LB medium. Cells were induced (ind.) with either 0.05% DCPK (pCOM10) or 1 mM IPTG (pBR22b) at the early exponential phase. After 4 h cells were processed and the fluorescence signal was determined. Error bars indicate standard deviations (n = 3).

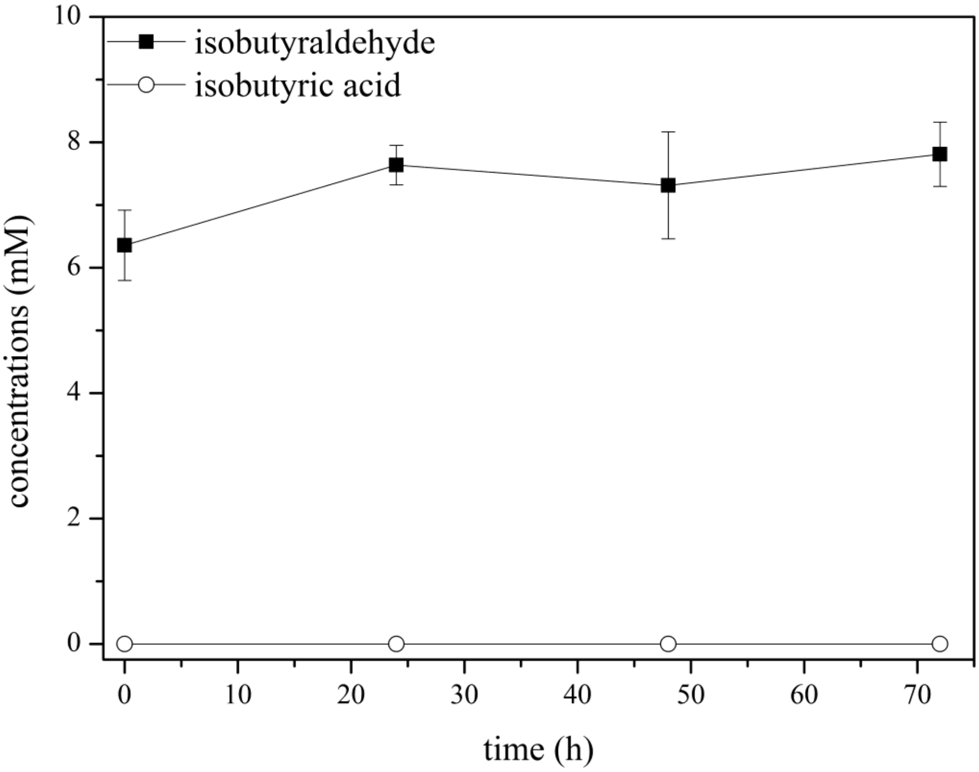

Figure S. 5. Abiotic incubation of 10 mM isobutyraldehyde in M9* medium pH 7.4 at 30°C. Fluctuation of isobutyraldehyde concentration is a result of high volatility of this compound. No formation of isobutyric acid could be observed. Error bars indicate standard deviations (n = 2).

Table S. 2. List of putative aldehyde dehydrogenases genes of *Pseudomonas* sp. strain VLB120

Number	Name	Gene product annotation	E.C. number
**PVLB_22545**	*ygaF*	hypothetical - hydroxyglutarate oxidase	--
**PVLB_18280**	*3821*		1.2.1.-
**PVLB_17530**	*astD*	N-succinyl-L-glutamate 5-semialdehyde dehydrogenase	1.2.1.-
**PVLB_12155**	*yneI*	aldehyde dehydrogenase family protein	1.2.1.-
**PVLB_26062**	*mhpF*	acetaldehyde dehydrogenase	1.2.1.10
**PVLB_07345**	*asd_1336*	aspartate-semialdehyde dehydrogenase	1.2.1.11
**PVLB_07340**	*asd_1335*	aspartate-semialdehyde dehydrogenase	1.2.1.11
**PVLB_22540**	*gabD*	succinate-semialdehyde dehydrogenase	1.2.1.16
**PVLB_01255**	*gabD*	succinate-semialdehyde dehydrogenase	1.2.1.16
**PVLB_22030**	*mmsA_3489*	methylmalonate-semialdehyde dehydrogenase	1.2.1.27
**PVLB_03765**	*mmsA_640*	methylmalonate-semialdehyde dehydrogenase	1.2.1.27
**PVLB_25000**	*5030*	4-guanidinobutyraldehyde dehydrogenase	1.2.1.3
**PVLB_24885**	*5008*	aldehyde dehydrogenase (NAD+)	1.2.1.3
**PVLB_18550**	*3875*	NADP-dependent aldehyde dehydrogenase	1.2.1.3
**PVLB_22390**	*aldB*	aldehyde dehydrogenase (NAD+)	1.2.1.3
**PVLB_12825**	*dhaS_2351*	aldehyde dehydrogenase family protein	1.2.1.3
**PVLB_26512**	*paaZ*	putative oxidoreductase	1.2.1.3
**PVLB_18550**	*3876*	NADP-dependent aldehyde dehydrogenase	1.2.1.36
**PVLB_11380**	*2630*	NADP-dependent aldehyde dehydrogenase	1.2.1.36
**PVLB_11105**	*dhaS_2005*	aldehyde dehydrogenase family protein	1.2.1.36
**PVLB_23045**	*argC_4661*	N-acetyl-gamma-glutamyl-phosphate reductase	1.2.1.38
**PVLB_10495**	*argC_1885*	N-acetyl-gamma-glutamyl-phosphate reductase	1.2.1.38
**PVLB_18510**	*3867*	ketoglutarate semialdehyde dehydrogenase/ NADP-dependent aldehyde dehydrogenase/2,5-dioxovalerate dehydrogenase	1.2.1.4
**PVLB_22325**	*proA*	glutamate-5-semialdehyde dehydrogenase	1.2.1.41
**PVLB_01470**	*calB*	coniferyl aldehyde dehydrogenase	1.2.1.68
**PVLB_21510**	*4455*	betaine-aldehyde dehydrogenase	1.2.1.8
**PVLB_19745**	*ydcW_4104*	gamma-aminobutyraldehyde dehydrogenase	1.2.1.8

**PVLB_10575**	*ydcW_1901*	gamma-aminobutyraldehyde dehydrogenase	1.2.1.8
**PVLB_10550**	*ydcW_1896*	gamma-aminobutyraldehyde dehydrogenase	1.2.1.8
**PVLB_01845**	*betB*	betaine aldehyde dehydrogenase	1.2.1.8
**PVLB_12010**	*2511*	putative glyceraldehyde-3-phosphate dehydrogenase	1.2.1.9
**PVLB_10355**	*1861*	putative aldehyde dehydrogenase	1.3.99.16

Table S. 3. List of putative alcohol dehydrogenases of *Pseudomonas* sp. strain VLB120

**Number**	**Name**	**Gene product annotation**	**E.C.number**
**PVLB_03315**		alcohol dehydrogenase, zinc-containing	
**PVLB_03955**		conserved hypothetical protein	
**PVLB_06715**		alcohol dehydrogenase, zinc-containing	
**PVLB_10545**		alcohol dehydrogenase, zinc-containing	
**PVLB_26027**	*adh*	alcohol dehydrogenase	1.1.1.1
**PVLB_26137**	*adh*	alcohol dehydrogenase	1.1.1.1
**PVLB_12675**	*attl*	putative alcohol dehydrogenase	1.1.1.1
**PVLB_11315**		cytochrome c family protein	
**PVLB_13895**		alcohol dehydrogenase, zinc-containing	
**PVLB_14065**		alcohol dehydrogenase, zinc-containing	
**PVLB_15055**		D-isomer specific 2-hydroxyacid dehydrogenase family protein	
**PVLB_15395**	*qor*	quinone oxidoreductase (NADPH:quinone reductase)	1.6.5.5
**PVLB_15440**	*adh5*	Alcohol dehydrogenase class-3	1.1.1.284
**PVLB_16075**	*adh2*	zinc-containing alcohol dehydrogenase superfamily	1.6.5.5
**PVLB_22260**	*adh*	2,3-butanediol dehydrogenase	
**PVLB_19365**		D-isomer specific 2-hydroxyacid dehydrogenase family protein	
**PVLB_23635**	*fdhA*	formaldehyde dehydrogenase, glutathione-independent	
**PVLB_24730**		alcohol dehydrogenase, zinc-containing	
**PVLB_00455**	*qor-1*	quinone oxidoreductase	
